# Correspondence on article by Pullakhandam et al. Reference cut-offs to define low serum zinc concentrations in healthy 1–19-year-old Indian children and adolescents

**DOI:** 10.1038/s41430-022-01152-z

**Published:** 2022-05-06

**Authors:** Christine M. McDonald, Mari S. Manger, Nancy F. Krebs

**Affiliations:** 1 Departments of Pediatrics, and Epidemiology & Biostatistics, University of California San Francisco, San Francisco, CA USA; 2 International Zinc Nutrition Consultative Group, San Francisco, CA USA; 3 Department of Pediatrics, University of Colorado School of Medicine, Aurora, CO USA

**Keywords:** Biomarkers, Signs and symptoms

## To the Editor:

In a recently published manuscript, Pullakhandam et al. summarize the results of an analysis that aimed to derive reference cut-offs to define low serum zinc concentrations (SZC) using data from presumably healthy children and adolescents 1–19 years of age who participated in India’s 2016–2018 Comprehensive National Nutrition Survey (ICNNS) [1]. The authors propose SZC cutoffs that are 10–18 μg/dL lower than those currently recommended by the International Zinc Nutrition Consultative Group (IZiNCG) [2, 3] and conclude that zinc deficiency is not a serious public health problem in any state or any child age group in India based on the application of these newly proposed cut-offs for Indian children.

The ICNNS has many strengths and is among the largest surveys to collect nationally-representative biochemical data on zinc status [4, 5]. The Indian nutrition community should be commended for this significant undertaking and it is encouraging to see many investigators make use of the rich dataset. However, there are significant technical limitations to Pullakhandam et al.’s approach and to the ICNNS SZC data that warrant careful consideration before drawing final conclusions.

A fundamental concern relates to the authors’ use of a statistical approach to derive cut-offs for deficiency based on a supposedly healthy subset of the Indian population. Although several sociodemographic, anthropometric, and biomarker filters were applied to exclude “unhealthy” children and adolescents, insufficient data are available to confirm that the remaining children in the various analytic samples were truly “healthy” with adequate dietary zinc intake and no clinical signs of zinc deficiency. Specifically, the primary analytic sample contains a considerable proportion of underweight and wasted children. Among children 1–5 years of age, 8.8% had a weight-for-age *Z* score < −2 and 9.3% had a weight-for-height *Z* score < −2; the prevalence of moderate or severe thinness according to BMI-for-age was 14.9% and 17.8% among children 5–9 years and adolescents 10–19 years of age, respectively. Although sensitivity analyses were conducted using primary analytic sample 2, which excluded children with severe underweight, wasting, and thinness, children with moderate forms of these conditions remained in the sample. Furthermore, while children with height-for-age Z scores (HAZ) < −2 were excluded from all analyses, the distribution of HAZ of children remaining in the samples was not reported and cannot be assumed to reflect that of a well-nourished population with a mean HAZ of 0. Thus, some of the children categorized as healthy had some degree of undernutrition and may have been at least mildly zinc deficient, which would result in a left-shifted distribution of SZC and a lower cut-off than desirable. The application of a lower reference cutoff means that some children with zinc deficiency will go unrecognized and will fail to benefit from appropriate interventions.

A second issue requiring attention is the results of the laboratory analysis of SZC. Defining the prevalence of low SZC at the population level is dependent on accurate and reasonably precise laboratory measurements of SZC. Although the authors describe the methods of internal quality control that were implemented as part of the analytical procedure, their reported coefficient of variation (CV) is 6.7%. This value is considerably higher than the overall CV of 2.1% reported in a recent study by Hall et al., which compared zinc measurements by Atomic Absorption Spectrometry, Inductively Coupled Plasma-Optical Emission Spectrometry, and Inductively Coupled Plasma-Mass Spectrometry in seven different laboratories around the globe [6]. The authors note that the ICNNS survey implemented “internal and external quality control procedures”, but without more data on the external quality control methods that were implemented, the accuracy of the SZC data should be interpreted with caution. It should be noted that a certified external quality assurance program does not currently exist for analysis of SZC and is desperately needed. The aforementioned study by Hall et al. reported inter-laboratory differences in calibration over 15% and outlines how such differences can impact prevalence estimates of zinc deficiency: in a hypothetical population of children with a mean SZC of 86 ± 13 μg/dL a 15% difference could cause the prevalence of SZC < 65 μg/dL to change from 2% to 17%.

The accompanying correspondence by Brown et al. summarize the benefits of using a single, internationally accepted cut-off, particularly for the purposes of monitoring the prevalence of zinc deficiency globally and allocating global resources. By investing in activities to improve the quality of data on zinc status, and reaching consensus on optimal laboratory methods and approaches to derive internationally acceptable reference values, the global nutrition community can make great strides to improve the quality of information on population zinc status and make more informed decisions on how best to ensure adequate zinc nutrition among vulnerable populations around the world.
